# CXCR7 Silencing Attenuates Cell Adaptive Response to Stromal Cell Derived Factor 1α after Hypoxia

**DOI:** 10.1371/journal.pone.0055290

**Published:** 2013-01-31

**Authors:** Sufang Liu, Xiaofeng Jia, Changsheng Li, Xuefei Han, Wenhai Yan, Ying Xing

**Affiliations:** 1 Department of Physiology, School of Medicine, Zhengzhou University, Zhengzhou, Henan Province, China; 2 Department of Biomedical Engineering, Johns Hopkins University School of Medicine, Baltimore, Maryland, United States of America; 3 Department of Physical Medicine and Rehabilitation, Johns Hopkins University School of Medicine, Baltimore, Maryland, United States of America; 4 Department of Anesthesiology and Critical Care Medicine, Johns Hopkins University School of Medicine, Baltimore, Maryland, United States of America; 5 Department of Anesthesiology, Henan Anti-cancer Hospital, Zhengzhou, Henan Province, China; Iowa State University, United States of America

## Abstract

Previous studies have shown that chemotactic factor stromal-cell derived factor 1α (SDF1α) promotes cell recovery from hypoxic injury via its main receptor C-X-C chemokine receptor type (CXCR) 4. However, the role of its new receptor CXCR7 on cell repair against hypoxia and cell response to SDF1α remains largely unknown. In this study, neurons induced from hippocampal progenitor cells were pre-conditioned in hypoxia for 4h and subsequently monitored to investigate the function of SDF1α on cell repair after hypoxia. Neurons were assessed for their cell morphology, actin filament polymerization and migration capability. SDF1α protein levels increased significantly 1 h after hypoxia compared to control (*P*<0.01), and it reached a peak at 24 h after hypoxia. Moreover, addition of SDF1α promoted neurite outgrowth and actin filament polymerization both in normoxic and hypoxic cells compared to untreated cells. Cell migration showed a time-dependent increase with SDF1α stimulation in both groups, and hypoxic cells illustrated a significant augment at 0.5 h, 1 h and 12 h after SDF1α application compared to normoxic cells (*P*<0.01). CXCR7 expression also increased with time dependence after hypoxia and demonstrated a two-fold upregulation compared to control at 24 h after hypoxia. With CXCR7 silencing, axon elongation and actin filament polymerization induced by SDF1α were inhibited sharply both in normoxic and hypoxic cells. CXCR7 silencing also leads to reduced hypoxic cell migration at 0.5 h, 1 h, 12 h, 24 h and 36 h after SDF1α application (*P*<0.01), but it failed to reduce normoxic cell migration induced by SDF1α at 0.5 h, 1 h and 12 h (*P*>0.05). 24 h SDF1α stimulation led to higher ERK1/2 phosphorylation compared to control, and ERK1/2 phosphorylation increased more in hypoxic cells than that in normoxic cells. This study suggested that CXCR7 plays an important role on cell repair processing induced by SDF1α, and CXCR7 silencing attenuates cell adaptive response to acute SDF1α stimulation (≤12 h) after hypoxia.

## Introduction

Previous studies have shown that neurons are able to withstand hypoxia by triggering endogenous survival and neuroprotective pathways [1]. These processes are always related to the recruitment of modulators that trigger some events promoting repair mechanisms [2], [3]. Recent studies have illustrated that a chemokine stromal cell derived factor 1α (SDF1α) is up-regulated in bone marrow mesenchymal stromal cells and synovial fibroblast cells to fulfill its role on cell protection against hypoxia [4], [5]. It increases neuron survival and protects them from hypoxia-induced cell death [6],[7]. Furthermore, neural chemokine SDF1α mediates axon elongation and neurite growth [8]. SDF1α may serve as a protective factor to promote cell repair after hypoxia, however, the exact effect of SDF1α on cell repair after hypoxia is still unclear.

Many studies have shown that SDF1α binds to C-X-C chemokine receptor type 4 (CXCR4) to exert its role on neurite outgrowth and cell migration [8], [9]. This view was challenged with the discovery that the orphan receptor RDC1, now designated as CXCR7 (C-X-C chemokine receptor type 7), is also able to bind SDF1α [10]. Many lines of evidence indicate that CXCR7 has an important role in regulating cell signaling in culture and in vivo. However, the function of CXCR7 in neurons toward SDF1α is under intense debate. While some reports have suggested that CXCR7 is necessary to regulate CXCR4 protein levels, thereby adapting chemokine responsiveness in migrating cells [11], other studies indicate that CXCR4 and CXCR7 have distinct roles and signal transduction in regulating interneuron movement and laminar positioning [12]. These results, along with similar observations in other tissues, led to the notion that CXCR7 modulates the effects of SDF1α by regulating chemokine gradients or counteracting with CXCR4 [13]. Nevertheless, analysis of CXCR7 function is limited to studies that demonstrate its function in normoxic culture conditions. The exact mechanisms involved in cellular response to SDF1α after hypoxia still require further investigation. Recently, CXCR7 has been shown to activate MAP kinases in transfected cells [14]. Additionally, a study showed that SDF1α mediates some signaling upon binding to CXCR7, leading to an increase in phosphorylation of ERK1/2 [15]. This leads to a plausible hypothesis that ERK1/2 may be involved in the cellular response to SDF1α-CXCR7 axis after hypoxic stimulation.

Here we have investigated the function of chemokine SDF1α on hippocampal cell repair after hypoxia by determining cell morphology, actin filament polymerization and migration capability. We found that CXCR7 silencing attenuates these processes by decreasing cell responsiveness to acute SDF1α stimulation. Moreover, ERK1/2 phosphorylation increased with SDF1α stimulation after hypoxia, whereas ERK1/2 phosphorylation decreased by pre-treatment with CXCR7 ShRNA. Thus, CXCR7 may trigger cell signaling in order to fulfill its role in modulating cellular response to SDF1α.

## Materials and Methods

### Ethics Statement

Sprague Dawley (SD) rats were provided by the Experimental Animal Center of Medical School at Zhengzhou University (Zhengzhou, China). This study was carried out in strict accordance with the Guidelines on the Care and Use of Laboratory Animals issued by the Chinese Council on Animal Research and the Guidelines of Animal Care. All procedures involving animals were approved by the Institutional Animal Care and Use Committees of Zhengzhou University. All efforts were made to minimize animals’ suffering and to reduce numbers of animals used.

### Cell Harvesting and Culture

Primary hippocampal progenitor cells were obtained from newborn SD rats as described previously [16]. Briefly, animals were euthanized under anesthesia. Bilateral hippocampi were carefully dissected on a cold stage and digested in 0.25% trypsin (Sigma-Aldrich) at 37°C for 5 min. Then the cells were collected and centrifuged at 1000 rpm for 5 min. The precipitant cells were gathered, washed and inoculated into culture plates at a density of 1×10^5^ cells/cm^2^ in serum-free DMEM/F12 medium supplemented with 2% B27, 20 ng/ml bFGF, 20 ng/ml EGF, kept in an incubator at 37°C in a 5% CO_2_ fully humidified atmosphere and fed half-fresh medium every 3 days. After about 7 days, the progenitor cells grew into ﬂoating neurospheres. These were mechanically separated into single cells and then passaged every 7 days. On passage 3, fetal bovine serum was added to the medium, and then the neurospheres adhered to the bottom of the culture dish and differentiated into neurons or glia-like cells. To further reduce growth of non-neuronal cells, cytosine arabinoside (AraC, 1 µM, Sigma-Aldrich) was added to the cultures within 24 h of plating. The single cells were incubated for 7 days to get the mature hippocampal neurons. On the seventh day of culturing, the neurons underwent hypoxia with 3% O_2_, 5% CO_2_ and 92% N_2_ for 4 h [17]. After the hypoxic period, the neuronal cells were returned to the original normoxic culture conditions at 37°C with 5% CO_2_ and 95% air to carry out experiments at different culture times.

### Neuronal Cell Count and Neurite Outgrowth Analysis

Neuronal cells were counted in 24-well culture dishes (six independent wells per time point) under a light microscope (Olympus, Japan). The photographed images were analyzed in a blinded fashion; the neuronal cells were identified by their typical morphology and counted manually. The number of neuronal cells per field was averaged to yield a cell number with standard deviation. The longest axon length and soma perimeter were quantitatively analyzed by microscope with Image-pro-Plus 5.0 software (Media Cybernetics, USA) [18].

### Enzyme-Linked Immunosorbent Assay (ELISA)

The production of SDF1α in the supernatants of hippocampal cells under normoxia or hypoxia was assessed by ELISA using a commercially available ELISA kit (R&D Systems, USA) according to the manufacturer’s recommendations. Supernatants were obtained and measured at 0.5, 1, 12, 24, and 36 hours after hypoxia.

### Migration Assay

Millicell hanging transwell chambers (Millipore, MA) with an 8-µm pore size polystyrene filter inserts were used according to the manufacturer’s instructions as described [19], [20]. Briefly, 1×10^5^ cells in serum-free DMEM/F12 medium were seeded into the upper compartment of each chamber, and serum-free medium with 50 ng/ml SDF1α was placed into the lower chamber. This optimal concentration was determined by a migration assay with normoxic cells stimulated by SDF1α at varying doses (1, 25, 50, 100 ng/ml) for 30 min (data not shown). Before migration testing, cells were divided into four parts with different treatments followed by SDF1α stimulation: (1) cells treated with hypoxia, (2) cells treated with CXCR7 ShRNA, (3) cells treated with CXCR7 ShRNA followed by hypoxia, and (4) cells left untreated. Cells seeded in the top chambers were incubated for 0.5, 1, 12, 24 and 36 hours with SDF1α stimulation, respectively. Then, non-migrating cells were removed from the top chamber, and migrated cells were fixed in methanol and stained with 2% toluidine. The number of cells that had migrated to the underside of the insert membranes was calculated by counting six random independent fields.

### Western Blotting

Adhered hippocampal neurons were washed with ice-cold PBS and scraped in RIPA lysis buffer including protease inhibitors. Total cell lysates were resolved on SDS-PAGE gels and transferred to a polyvinylidene difluoride membrane. SDF1α, CXCR7 (1∶2000, Abcam), GAPDH, phospho-ERK and total-ERK antibodies (1∶1000, Beyotime Institute of Biotechnology, China) were used for the analyses. Six independent experiments were applied for western blot. Density analysis of protein bands were calculated by Image J 1.4 software, and each band was normalized with the integrated density of the corresponding reference band [21].

### Immunocytochemistry

Briefly, adhered hippocampal cells were fixed for 30 min with 4% paraformaldehyde. Nonspecific binding was blocked with 2% bovine serum albumin (BSA) for 1 h, followed by incubation with CXCR7 primary antibody overnight at 4°C (1∶100, Abcam). Cells were then incubated with fluorescently labeled rabbit secondary antibody (Sigma, 1∶200) for 1 h respectively. CXCR7 positive cells of six fields of each independent experiment were counted manually under a light microscope (Olympus, Japan). The number of positive cells per field was averaged to yield a cell number with standard deviation. Alexa Fluor 546-conjugated phalloidin was used as a high-affinity F-actin probe (Invitrogen, USA).

### Lentiviral Transduction of Hippocampal Cells

Three different short hairpin RNA (ShRNA) sequences directed against CXCR7 were purchased from Shanghai GenePharma (China). Three ShRNA lentiviral expression vectors with green fluorescent protein (GFP) were transfected into hippocampal cells targeting CXCR7 mRNA (GenBank NM_053352.1) sequence. To make the constructs, a BbsI site was added to the 5′ end of the ShRNA sequence and a XohI site to the 3′ end. Three ShRNA sequences that target the rat CXCR7 sequence were designed as follows: 5′-GGAACTACTCGGACATCAACT-3′ (sense) and 5′-AGTTGATGTCCGAGTAGTTCC-3′ (antisense, CXCR7ShRNA1); 5′-GGGTGAATATCCAGGCCAAGA-3′ (sense) and 5′-TCTTGGCCTGGATATTCACCC-3′ (antisense, CXCR7 ShRNA 2); 5′-GGTCAGTCTCGTGCAGCATAA-3′ (sense) and 5′-TTA TGCTGCACGAG ACTGACC-3′ (antisense, CXCR7 ShRNA 3). The lentivirus-GFP that expressed GFP only was used as a blank control. On the seventh day of culturing, some of transfected cells were assessed via western blot to detect expression of the CXCR7 gene, while others were exposed to hypoxia as described above. After the hypoxic period, the neuronal cells were returned to the original normoxic culture to carry out the experiments for different culture periods.

### Statistical Analysis

Statistical analysis was performed using a standard computerized statistical package (Statistics Program for the Social Sciences version 16.0, Chicago IL). Parametric data are expressed as the mean ± standard deviation of each group. Analysis of variance (ANOVA) was performed for parametric data with the use of least significant difference (LSD) analysis used for multiple comparisons. An alpha level <0.05 was selected to consider the differences significant.

## Results

### Expression of SDF1α in Cultured Hippocampal Cells after Hypoxia

**Figure 1:**
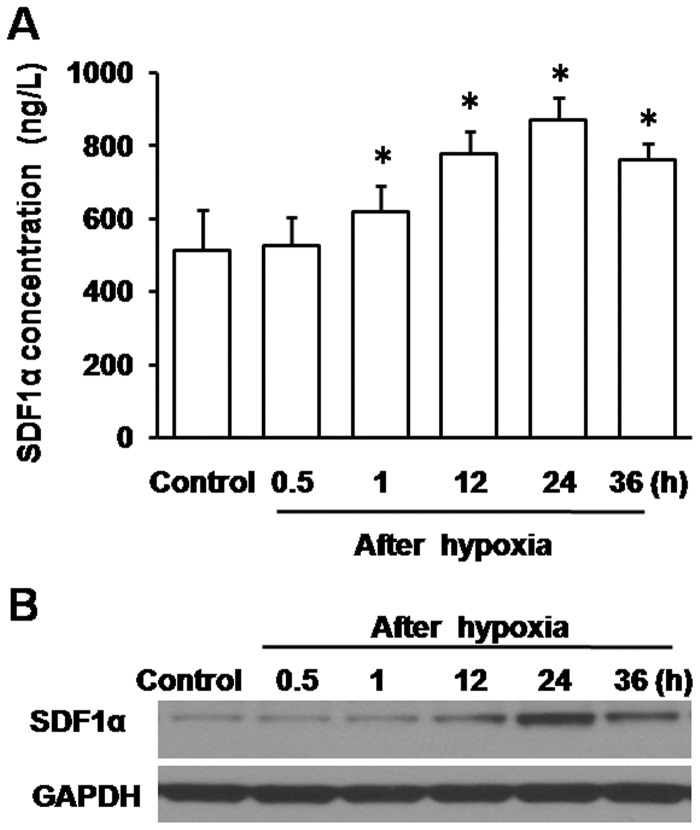
Expression of SDF1α in hippocampal cells after hypoxia. Adhered hippocampal cells were exposed to hypoxia (3%O_2_, 5% CO_2_ and 92% N_2_) for 4 h or left untreated on the seventh day of culturing. Supernatants and cell lysates were obtained and measured at 0.5, 1, 12, 24, and 36 h after hypoxia for ELISA and western blot respectively. Untreated cells were used as control. A, Concentrations of supernatant SDF1α in the medium were determined by ELISA. *P<0.01, vs control. B, Expression of SDF1α in the cells was measured by western blot analysis. GAPDH was used as reference.

Expression of SDF1α in cultured hippocampal cells at 0.5, 1, 12, 24, and 36 h after hypoxia is shown in Figure 1A. Comparison of the expression level of SDF1α at different culture stages revealed that SDF1α secreted in the medium was increased to 618.65±70.46 ng/L at 1 h after hypoxia compared to control (513.94±107.76 ng/L, *P*<0.01). It reached peak levels at 24 h followed by a decrease at 36 h (*P*<0.01), which may be contributed to neural cells binding and taking up secreted SDF1α in the medium. However, analysis of protein expression in the cells (Figure 1B), revealed an up-regulation of SDF1α at 12 h after hypoxia, most likely due to synthesis of SDF1α in the cytoplasm. Thus, hypoxic pre-conditioning leads to an increase of SDF1α expression in both secreted and synthesized forms.

### Effects of SDF1α on Cell Morphology, Actin Filament Polymerization and Migration Capability after Hypoxia

**Figure 2:**
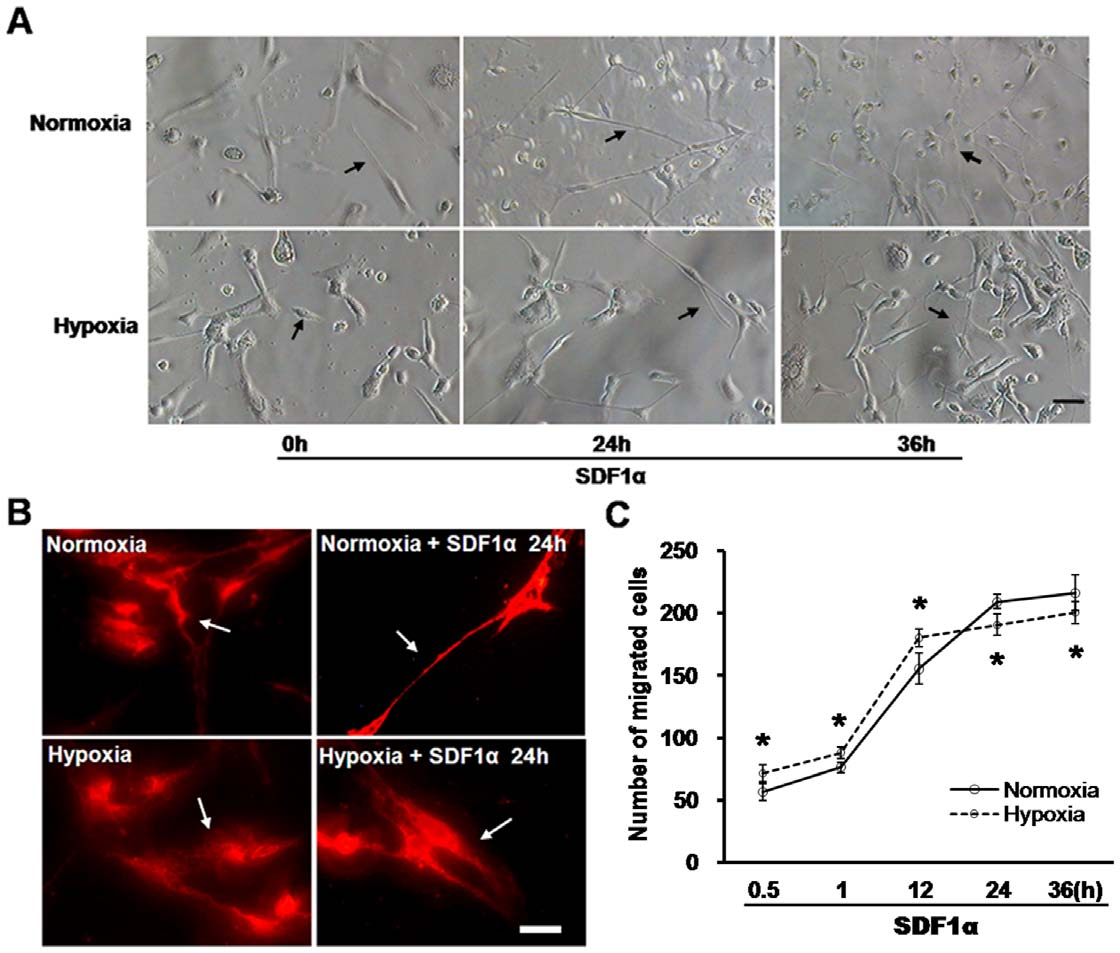
Effects of SDF1α on cell morphology, actin filament polymerization and migration capability after hypoxia. Adhered hippocampal cells were exposed to hypoxia or left untreated (normoxia) on the seventh day of culturing. Then SDF1α (50 ng/ml) was added to medium for 0.5, 1, 12, 24, and 36 hours. A, Cell morphological changes were observed in normoxia and hypoxia with 24 or 36 h stimulation of SDF1α or not. Arrow heads indicate the changes of morphology. Bar = 50 µm. B, Actin filament polymerization was measured by Alexa Fluor 546-conjugated phalloidin under normoxia or hypoxia with SDF1α for 24 h or not. Distribution changes of actin filament polymerization are indicated by arrows. Bar = 50 µm. C, Cell migration induced by SDF1α was determined by a transwell chamber assay. *P<0.01, vs normoxia.

Cells treated with hypoxia conditions displayed an overall decrease in dendrite length and shorter branches compared with the normoxia group (shown by arrows). However, application of SDF1α for 24 or 36 hours almost completely repaired cell morphologies including neurite outgrowth and neural network, which were initially damaged in the early stages after hypoxia (Figure 2A). In addition, 24 h SDF1α stimulation increased actin filament polymerization in axons and dendrites both in normoxic and hypoxic cells (Figure 2B), but not in soma (Detail data shown in Table S1).

As shown in Figure 2C, SDF1α enhanced cell migration with time dependence from 0.5 h to 36 h both in normoxic and hypoxic cells. The number of migrated cells in the hypoxic group accounted for 71.50±6.60, showing a significant increase compared to normoxia group (56.5±6.95) with stimulation of SDF1α for 0.5 h (*P*<0.01). Stimulation with SDF1α resulted in a robust migratory response of both normoxic and hypoxic pre-conditioned cells, but with differences in timing of the response. In the first 12 h of SDF1α treatment, cell migration was significantly higher in the hypoxic pre-conditioned group (180.17±12.40) versus normoxia (155.33±7.12, *P*<0.01). However, after 24 h exposure of SDF1α, cell migration of normoxic cells accounted for 209.33±8.55, greater than hypoxic cells (190.67±5.57, *P*<0.01). And 36 h SDF1α application showed a significant increase of migrated cells in normoxic group (216.17±8.98) than hypoxic group (200.33±14.3, *P*<0.01).Together with observations above, these results strongly suggest that cells pre-conditioned in hypoxia showed higher sensitivity to respond to acute SDF1α stimulation (≤12 h) compared with that of normoxic cells.

### Expression of CXCR7 in Cultured Hippocampal Cells after Hypoxia

**Figure 3:**
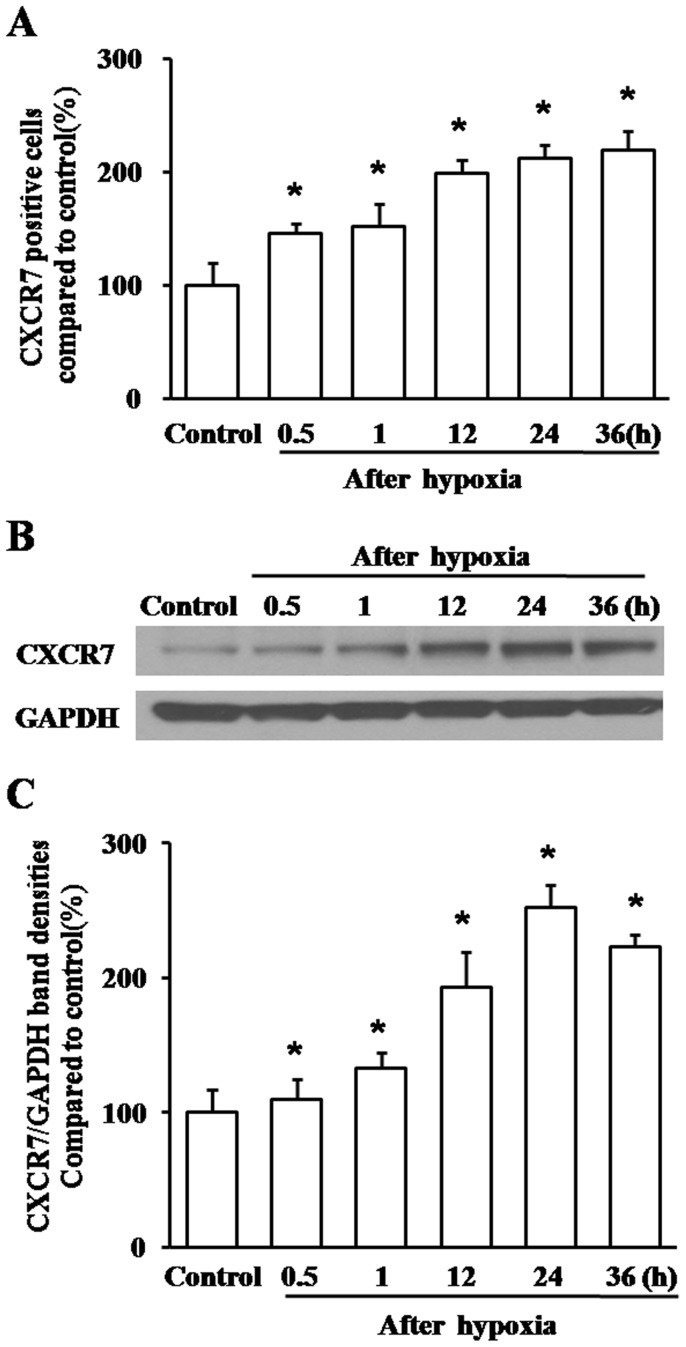
Expression of CXCR7 in hippocampal cells after hypoxia. Adhered hippocampal cells were exposed to hypoxia or normoxia (control), and then CXCR7 expression was determined by immunofluorescence and western blot at 0.5, 1, 12, 24, and 36 h after hypoxia. Six independent experiments were applied for both cell count and western blot. A, Hippocampal cells were digested from the culture dish and measured for CXCR7 expression by immunofluorescence. *P<0.01, vs control. B–C, Cell lysates were obtained to perform western blot assay for CXCR7 expression. Data showed strong up-regulation of CXCR7 after hypoxia. *P<0.01, vs control.

The number of CXCR7 positive cells increased in a time-dependent manner after hypoxia (Figure 3A) to 85.67±4.46 at 24 h, which was two times greater than control (40.33±7.76, *P*<0.01). Measurement of western blot confirmed the above mentioned results, showing a significant increase in CXCR7 expression 24 h after hypoxia, the CXCR7 expression was doubled compared to control measurement (Figure 3B–C, *P*<0.01). Conversely, a decline of CXCR7 expression 36 h after hypoxia was observed relative to that of 24 h, which can probably be attributed to combination with its ligand, SDF1α (Figure 3B–C, *P*<0.01).

### Effects of CXCR7 ShRNA on Cell Morphology, Actin Filament Polymerization and Migration Capability Induced by SDF1α in Normoxic Cells

**Figure 4:**
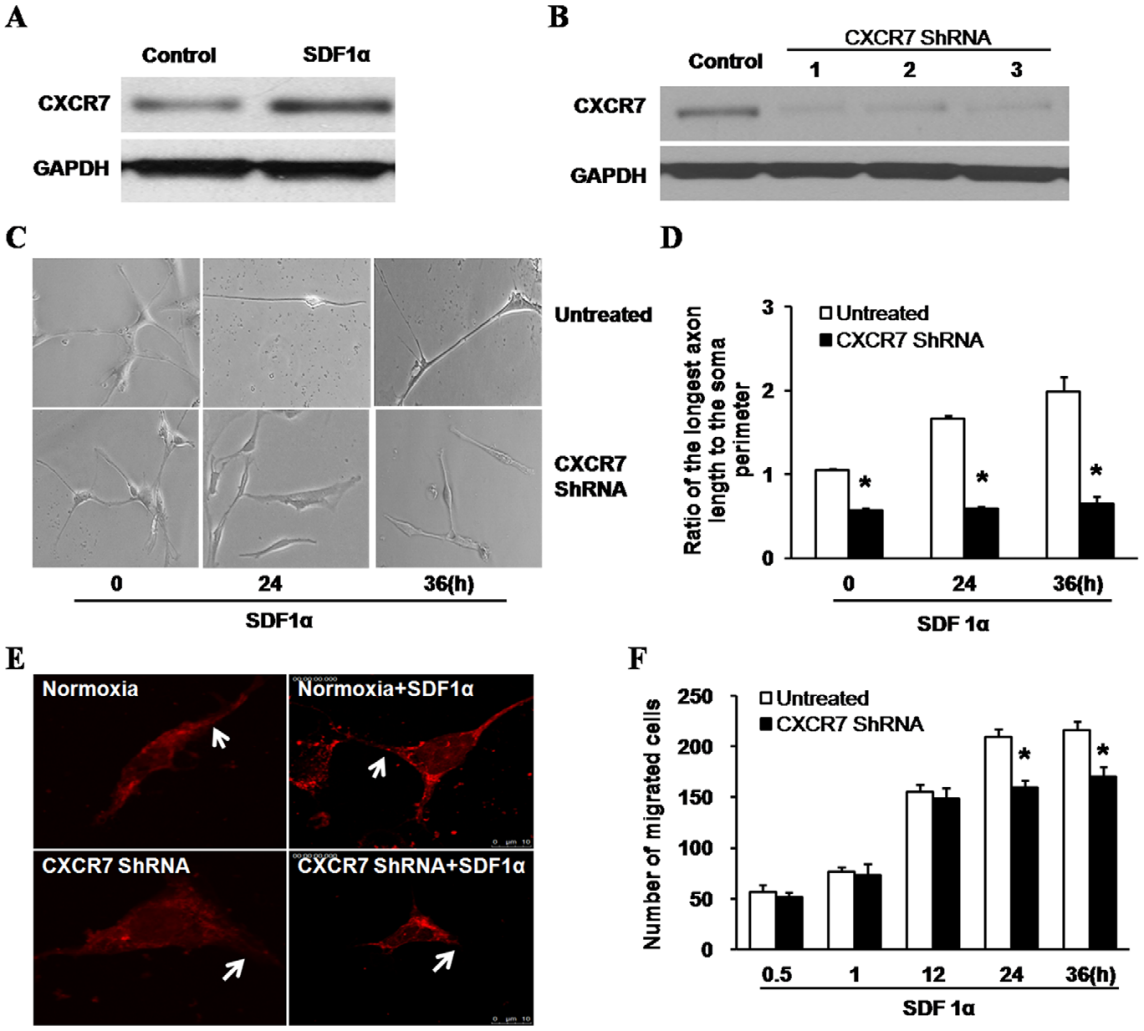
Effects of CXCR7 ShRNA on cell morphology, actin filament polymerization and migration capability induced by SDF1α in normoxic cells. A, CXCR7 expression was increased with 24 h stimulation of SDF1α. Adhered hippocampal cells were cultured in normoxia for seven days. Then cells were treated with SDF1α (50 ng/ml) for 24 h or left untreated (control). Cell lysates were obtained to detect CXCR7 expression by western blot. B, CXCR7 protein expression was determined after CXCR7 silencing. Three ShRNA (CXCR7 ShRNA1, CXCR7 ShRNA2, CXCR7 ShRNA3) lentiviral expression vectors with green fluorescent protein (GFP) were transfected into hippocampal cells targeting CXCR7 mRNA on the 7^th^ day of culture. Three days later, cell lysates of transfected and untreated cells (control) were obtained to determine CXCR7 protein expression by western blot. The lentivirus-GFP that expressed GFP only was used as a blank control (data was not shown). Here, we used CXCR7 ShRNA1 as an effective inhibitor to do subsequent experiments. C, D, Cell morphology was observed and the ratio of the longest axon length to the soma perimeter was calculated by image analysis system. Cells were cultured under normoxia with or without CXCR7 ShRNA transfection followed by SDF1α (50 ng/ml) for 24 or 36 hours, or left untreated. Neuronal cells were counted under a light microscope. Six predetermined areas in each independent well (From a total of six wells) at each time point were selected and photographed. Then the longest axon length and the soma perimeter were counted and calculated manually. Bar = 10 µm. *P<0.01, vs untreated. E, Actin filament polymerization was observed by staining with phalloidin and imaging with confocal microscopy. Neural cells were transfected with CXCR7 ShRNA with following SDF1α (50 ng/ml) application for 24 h or not. Arrows indicate the distribution changes of actin filament polymerization. Bar = 10 µm. F, Number of migrated cells was counted by transwell chamber analysis. Some of cells were transfected with CXCR7 ShRNA, and some were left untreated. Then cells were placed in the upper chamber and SDF1α (50 ng/ml) was added to the lower chamber for 0.5, 1, 12, 24, and 36 hours respectively for migration analysis. *P<0.01, vs untreated.

To study whether CXCR7 was involved in cell repair processes induced by SDF1α, CXCR7 expression was firstly detected in cultured cells treated with 24 h SDF1α application (Figure 4A), and western blotting illustrated a strong increase of CXCR7 expression compared to untreated cells. Then three ShRNA were used to knock down CXCR7 expression, each of which was effective in inhibiting CXCR7 protein expression (Figure 4B). Here, we used CXCR7 ShRNA1 as an effective inhibitor to do following experiments. As shown in Figure 4C–D, the ratio of the longest axon length to the soma perimeter after CXCR7 silencing was 0.57±0.02, showing almost half decline compared to control (1.06±0.02). Following 12 h and 24 h SDF1α stimulation, the ratio in normoxic cells increased to 1.67±0.03 and 1.99±0.18, showing significant increase than that of cells with CXCR7 silencing (0.59±0.03 and 0.65±0.09) respectively.

CXCR7 was then tested for its involvement in recombination of cytoskeletons like actin filament polymerization. As shown in Figure 4E, actin filament polymerization was mainly distributed in the soma in both normoxic and CXCR7 ShRNA exposed cells without SDF1α application. However, after 24 h SDF1α stimulation, actin polymerization was increased and redistributed in neurites and sprouts in normoxic cells, but not in cells with CXCR7 silencing (shown by arrows).

Unexpectedly, as shown in Figure 4F, there was not a significant difference in the number of migrated cells between the untreated group and CXCR7 ShRNA group with 0.5 h, 1 h, 12 h SDF1α stimulation respectively (*P*>0.05). The number of migrated cells in the untreated group was 56.50±6.95 after 0.5 h application of SDF1α. However, unlike groups with acute SDF1α exposure (≤12 h), migrated cells in CXCR7 ShRNA group (159.67±7.34) still illustrated a decline compared to untreated group (209.33±8.55) after 24 h SDF1α application.

### Effects CXCR7 ShRNA on Cell Morphology and Migration Capability Induced by SDF1α in Hypoxic Cells

**Figure 5:**
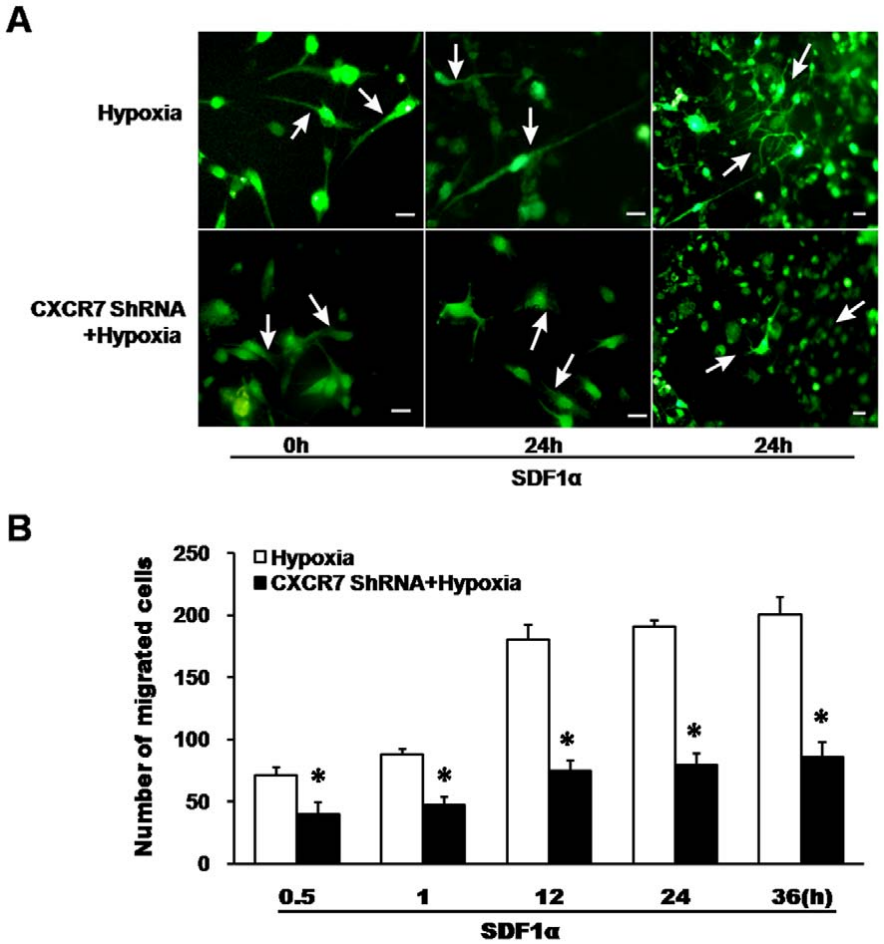
Effects of CXCR7 ShRNA on cell morphology and migration capability induced by SDF1α in hypoxic cells. A, Cell morphology was observed after hypoxia with pre-treatment of CXCR7 ShRNA or not. Some of cells were transfected with CXCR7 ShRNA, or left untreated. Then all of cells were treated with hypoxia followed by stimulation of SDF1α (50 ng/ml) for 24 h. Neuronal cell morphology was observed and photographed under a fluorescence microscope. Arrows indicate the changes of dendrite length and neurite outgrowth formation. Bar = 30 µm. B, Number of migrated cells was counted by transwell chamber analysis. Cells with or without CXCR7 silencing were treated with hypoxia, and then were placed in the upper chamber with SDF1α (50 ng/ml) in the lower chamber for 0.5, 1, 12, 24, and 36 hours respectively. *P<0.01, vs hypoxia.

As shown in Figure 5A, cells with CXCR7 ShRNA exposure followed by hypoxia showed less neurites and shorter axons compared to those treated with hypoxia only, following 24 h stimulation of SDF1α (shown by arrows). Then cell migration was determined with 0.5 h, 1 h, 12 h, 24 h and 36 h SDF1α stimulation as shown in figure 5 B. The number of migrated cells induced by 0.5h SDF1α application was significantly greater in the group that was exposed to hypoxia (71.50±6.60) compared to the group in which hypoxia was preconditioned by CXCR7 ShRNA (39.50±10.21, *P*<0.01). The difference still was apparent in two groups at 36 h after SDF1α application (200.33±14.39 and 85.83±12.01, respectively, *P*<0.01). In addition, number of cell migration showed a stronger upward tendency in the hypoxic group than that of CXCR7 ShRNA-hypoxia group with time-dependent stimulation of SDF1α.

### Expression of ERK1/2 Phosphorylation Induced by SDF1α after Hypoxia with CXCR7 Silencing

**Figure 6:**
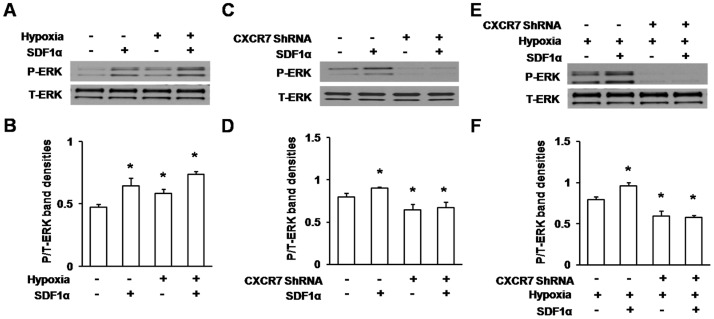
Expression of ERK1/2 phosphorylation induced by SDF1α after hypoxia with CXCR7 silence. Cell lysates were obtained with different treatment to detect phosphorylated ERK1/2 (P-ERK) and total ERK1/2 (T-ERK) by western blot. Total ERK was used as a control. A–B, Detection of ERK phosphorylation induced by hypoxia and/or SDF1α was performed. Cells were treated with hypoxia and/or SDF1α (50 ng/ml) for 24 h, or left untreated. *P<0.01 vs untreated group. C–D, Detection of ERK1/2 phosphorylation induced by SDF1α after CXCR7 silence was performed. Cells were transfected with CXCR7 ShRNA and/or with application of SDF1α (50 ng/ml) for 24 h, or left untreated. *P<0.01 vs untreated group. E–F, Detection of ERK1/2 phosphorylation induced by SDF1α after CXCR7 silencing and hypoxia was performed. Cells were transfected with CXCR7 ShRNA or not, then all of cells were treated with hypoxia with presence or absence of SDF1α (50 ng/ml) for 24 h. *P<0.01 vs group with hypoxia exposure.

As shown in Figure 6, ERK1/2 phosphorylation was detected with different treatments. We observed that 24 h SDF1α stimulation strongly caused ERK1/2 phosphorylation, which was remarkably significant in cells pre-conditioned with hypoxia compared to untreated group (Figure 6A–B, *P*<0.01). Then we found cells transfected with CXCR7 ShRNA failed to increase phosphorylated ERK1/2 expression with SDF1α stimulation for 24 h compared to untreated group (Figure 6C–D, *P*<0.01). Though ERK1/2 phosphorylation induced by SDF1α increased significantly in hypoxic cells, it was still inhibited following treatment with CXCR7 ShRNA(Figure 6E–F, *P*<0.01). Altogether, our experiments demonstrated that ERK1/2 pathway was activated by hypoxia and SDF1α/CXCR7 pathway.

## Discussion

In this study, we have used hippocampal cells cultured in vitro as a model system to investigate the function of atypical chemokine receptor CXCR7 in cellular response to SDF1α after pre-conditioning in hypoxia. We found that CXCR7 is required in hippocampal cells to regulate cell adaptive functions with chemokine SDF1α stimulation, including cell morphology, actin filament polymerization and migration capability. However, these functions emerge as significant properties of hypoxic cells, because the interference of CXCR7 altered cell migration ability in hypoxic cells much more than in normoxic cells. These results provide a clear demonstration that an atypical chemokine receptor can modulate cell response to classical chemokine SDF1α after hypoxia.

Our results showed that SDF1α was highly up-regulated in cultured hippocampal cells after hypoxia, which is consistent with previous studies findings that SDF1 is over-expressed in inflammatory processes in several brain diseases with some neuroprotective function [4], [5], [20], [22]. However, a study demonstrated that SDF1α expression in neural progenitor cells derived from neonatal mouse brain showed no response to chronic hypoxia [22], [23]. Thus, SDF1α seems to be involved in cell adaptive changes to acute hypoxia but not to chronic hypoxia [24].

Our results have shown that 24 h application of SDF1α repaired neurite outgrowth and neural network damaged by hypoxia. We also found that SDF1α may change or recombine cell cytoskeleton by increasing actin filament polymerization, thus resulting in cell adaptive survival after hypoxia. This is consistent with previous studies that SDF1α improves neurite outgrowth and axon elongation in corticospinal tract axons and cultured cerebellar granule neurons [25], [26]. Drastic changes in neuronal shape occur immediately after the exit of neuronal cells from mitotic cycles during embryonic and postnatal development. Thus, it is conceivable that SDF1α might be necessary for reparative capabilities after hypoxia as a mitogen and potential target for morphogenesis and neuroprotective strategies.

Previous studies have shown that SDF1α increases the motility of upper rhombic lip cells and promotes the migration of granule cell progenitors and interneurons [27], [28]. However, whether SDF1α could affect cell migration under hypoxic conditions still needs further investigation. A recent study illustrated that SDF1α could direct the migration of neural progenitor cells under hypoxic circumstances [29], and guide migration after focal cerebral ischemia [30]. Our observations suggested that SDF1α enhanced cell migration especially when cells were treated with hypoxia. Though our observations provided evidence that cells have a high sensitivity to respond to SDF1α at early stages after hypoxia, we still wonder if it is hypoxia or SDF1α that mainly increased cell migration.

It has been shown that CXCR7 is expressed in most neurons of human brain cultures [31]. In the embryonic rat cortex numerous cells also express CXCR7, which is found to be very abundant in neurons forming the cortical plate during initial stages of corticogenesis [11], [12]. In our work we found CXCR7 was expressed in hippocampal cells and was increased significantly with application of SDF1α for 24 hours. Indeed, CXCR7 is evenly distributed in DRG neurons both at early and at late stages of cultivation [8]. Moreover, previous studies have suggested that hypoxic preconditioning increases CXCR7 expression in mesenchymal stromal cells [32], and only CXCR7 activity was functionally linked to survival signaling in mouse neural progenitor cell cultures during hypoxia exposure [33]. In our experiments, both CXCR7 positive cells and CXCR7 protein expression increased at 12 h after hypoxia. CXCR7 expression in the brain has been shown to increase after stroke [34], and seems to exert a prominent role in the immediate regulation of cell transduction pathways [35]. Thus it is possible that CXCR7 might be necessary for cell adaptive functions after hypoxia.

Our work showed no significant difference between untreated cells and CXCR7 ShRNA transfected cells with acute stimulation of SDF1α, but a remarkable difference was shown after 24 h application of SDF1α. This result was partly consistent with a study in which CXCR7 is involved in neuronal cell migration of rat forebrain, independent of the main receptor CXCR4 [36], and CXCR7 mutants also show a reduced rate of interneuron migration and laminar positioning defects [12]. This suggests that CXCR7 may be necessary, but not sufficient for cell migration, as pre-treatment of CXCR7 ShRNA could not block the time-dependent promotion of SDF1α until 24 hours later.

Indeed, CXCR7 was necessary for survival signaling in mouse neural progenitor cells during hypoxic exposure. A recent study suggests that down-regulation of CXCR7 results in reduced interneuron responsiveness to SDF1α [12]. Our observations showed that CXCR7 silencing caused neurite outgrowth defects and migration capacity decline significantly. Taken together with previous data, we found that hypoxia increased cell responsiveness to acute SDF1α stimulation, but this responsiveness was abrogated by pretreatment with CXCR7 ShRNA. These results mentioned above strongly suggested that CXCR7 was critical and required in cell response to available SDF1α, thereby modulating neurite outgrowth and cell migration.

The ERK1/2 signaling pathway is critical for cell survival and migration. Previous studies have shown that Raf/ERK up-regulation enhances the migration of cultured cortical and cerebellar granule cells [33], and that the ERK1/2 pathway is involved in neuronal differentiation, synaptic plasticity and cell migration [37], [38]. As a mitogen and chemokine, SDF1α induces ERK1/2 activation in activated T cells [39]. Our results, consistent with these studies, also showed a significant increase of ERK1/2 phosphorylation induced by 24 h SDF1α stimulation.

A recent study showed that hypoxia induced ERK1/2 phosphorylation [40], [41], which is involved in mitochondrial biogenesis in hippocampal cells during hypoxia exposure [42], [43]. In our work, we also found phosphorylated ERK1/2 increased significantly after hypoxia. Stimulation with SDF1α strongly promotes the phosphorylation of ERK1/2 in migrating interneurons, but fails to elicit phosphorylation of ERK1/2 in cells obtained from CXCR7 mutants [11]. Thus we wondered whether CXCR7 was involved in ERK1/2 phosphorylation activated by SDF1α after hypoxia. Our data using cultures of hippocampal cells supported this hypothesis. We found that ERK1/2 phosphorylation was inhibited after CXCR7 silencing, either with treatment of hypoxia or SDF1α stimulation. Thus, it is conceivable that SDF1α, acting through its receptor CXCR7, increased cell adaptive function via activation of ERK1/2.

We believe that our findings may have important implications to repair cell function after hypoxia. CXCR7 has been involved in multiple steps induced by SDF1α stimulation, including neurite outgrowth, actin filament polymerization and cell migration. If one important function of CXCR7 is to alter cell response toward SDF1α, as reported here, then modulating this receptor may contribute to repair activities from hypoxia. While we show effects between control cultures in approximately 20% oxygen and the hypoxia treated cells, however, caution must be exercised in extending the observations to in vivo responses to low oxygen levels (e.g. 3% Oxygen), which is close to physiological levels within tissues of an intact organism.

## Supporting Information

Table S1 **Fluorescence mean densities of actin filament polymerization.** OD: optical density. *P<0.05, vs normoxia; #*P*<0.05, vs hypoxia.
(PDF)
