# Supplementary material for: CXCR7 Silencing Attenuates Cell Adaptive Response to Stromal Cell Derived Factor 1α after Hypoxia
Source: PLoS One. 2013 Jan 31;8(1):e55290. doi: 10.1371/journal.pone.0055290 (PMC3561379; doi:10.1371/journal.pone.0055290)
Supplement: Table S1 — Fluorescence mean densities of actin filament polymerization. OD: optical density. *P<0.05, vs normoxia; #P<0.05, vs hypoxia. (PDF) [file pone.0055290.s001.pdf]

Table S1 Fluorescence mean densities of actin filament polymerization

	Dendrites (OD)	Soma (OD)
Normoxia	18.71±4.08	34.42±12.97
Normoxia+SDF1α	24.70±6.68[*]	35.89±11.41
Hypoxia	13.80±5.53	24.98±9.28
Hypoxia+SDF1α	22.21±8.43[#]	26.98±8.52

*$P<0.05$, vs normoxia; #$P<0.05$, vs hypoxia.
